# Oil, Gas and Conflict: A Mathematical Model for the Resource Curse

**DOI:** 10.1371/journal.pone.0066706

**Published:** 2013-06-27

**Authors:** Yiyong Cai, David Newth

**Affiliations:** 1 CSIRO Centre for Complex Systems Science, Commonwealth Scientific and Industrial Research Organisation, Canberra, ACT, Australia; 2 Centre for Applied Macroeconomic Analysis, Australian National University, Canberra, ACT, Australia; Universidad Carlos III de Madrid, Spain

## Abstract

Oil and natural gas are highly valuable natural resources, but many countries with large untapped reserves suffer from poor economic and social-welfare performance. This conundrum is known as the resource curse. The resource curse is a result of poor governance and wealth distribution structures that allow the elite to monopolize resources for self-gain. When rival social groups compete for natural resources, civil unrest soon follows. While conceptually easy to follow, there have been few formal attempts to study this phenomenon. Thus, we develop a mathematical model that captures the basic elements and dynamics of this dilemma. We show that when resources are monopolized by the elite, increased exportation leads to decreased domestic production. This is due to under-provision of the resource-embedded energy and industrial infrastructure. Decreased domestic production then lowers the marginal return on productive activities, and insurgency emerges. The resultant conflict further displaces human, built, and natural capital. It forces the economy into a vicious downward spiral. Our numerical results highlight the importance of governance reform and productivity growth in reducing oil-and-gas-related conflicts, and thus identify potential points of intervention to break the downward spiral.

## Introduction

**Figure 1:**
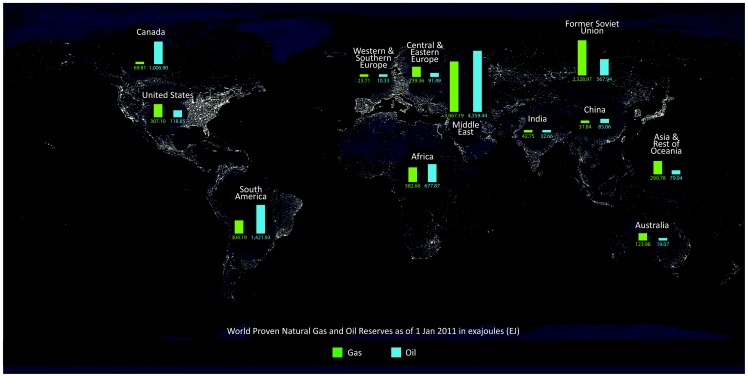
World's Proven Oil and Gas Reserves, and Earth's City Lights. Background image courtesy of NASA and data courtesy of CIA-The World Factbook.

Oil and gas are common, high-value commodities in the world market. They are also essential commodities for economic growth and development. Prices for oil and gas have increased dramatically over the last few decades and are expected to continue to do so. Industrial processes, such as electricity generation, machine operation, and petroleum chemical production, require oil and gas. Therefore, areas with abundant oil and gas reserves should be prosperous; however, economists have shown that oil-and-gas-rich countries usually suffer from poor economic performance. The few exceptions include Australia, Canada, and Norway, which are all countries with a democratic regime and a workable tax system that redistributes profits from mining to the rest of the economy and that sustains peaceful development. This economic phenomenon is referred to as the *resource curse* [1], [2]. Moreover, energy consumption per capita is often far below the world average in oil-and-gas-rich countries, although these exports constitute most of the countries economies (see Figure 1). This is further referred to as the *poverty in the midst of plenty*.

Research on this resource development puzzle tends to focus on oil-and-gas-related civil conflict. The high value and high utility of oil and gas make them points of contest among different social groups. In a weak government, greedy elite may appropriate national patrimony to advance their personal fortunes, while frustrated civilians may use violence to gain control over oil and gas resources. In turn, the elite resort to outright repression to keep the civilians in check. The subsequent escalation of the attack-and-defence cycle displaces human, built, and natural capital [3]–[5]. It also generates political instability, which depresses investment and impedes economic growth [6]–[8]. Therefore, despite years of oil and gas extraction, a resource-rich country in civil conflict remains underdeveloped with an economy that is dangerously reliant on oil and gas exports [9]–[12]. This instability intensifies political competition for control over oil and gas reserves and gives rise to a loop of causalities between resource dependence and conflict [13].

Political economy models generally consider conflicts to be equilibrium behaviors of different interest groups. These models commonly assume that the opportunity costs of attack and defence, or equivalently the productive returns on resources and labor, are exogenously given [3], [14]–[16]. However, these conflict models are insufficient to address the resource curse. It is plausible that a resource-abundant country in conflict is worse off than it is in the absence of conflict [17]. Nevertheless, it is implausible that a country is worse off than it would be without its natural resources, simply because it could neglect its resources and thereby escape from the curse. Therefore, particular attention must be given to the underlying institutions that drive the economy into self-destruction, such as social fractionalization [18]. Furthermore, reduced-form regressions based on these models may be subject to the problem of endogeneity, because of the possible causality loop between oil dependence and conflict. Subsequently, these regressions produce biased estimators, unless a natural experiment is available with relevant content, such as the discovery of an oil field and the subsequent civil conflict. This poses a challenge for empirical studies of the mechanisms that underlie the resource curse and for the formation of related policies.

This paper offers a supplementary perspective to the current understanding of the resource curse by using the context of oil, gas, and conflict. It relates poor economic performance to the existence of social fractionalization (elite and civilian), market frictions (monopolistic resource pricing), and resource-related conflict (economic disturbance). When oil and gas are monopolized by the elite, they are often exported rather than sold domestically to support local production. Increased exportation lowers the marginal return to productive activity, and consequently, civil insurgency emerges. The resultant conflict further displaces resources and labor and thus draws the economy into a vicious circle. In the absence of a natural experiment, this research provides a potential alternative structure for econometric identification of the mechanism that drives the resource curse. Additionally, it offers guidance to international organizations on the formation of policies for conflict resolution and poverty reduction.

## Analysis

### Background

We consider a two-period game that is set up in a small, open economy. The economy has two sectors: extraction and production. The game lasts for two periods 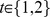. Let 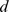 be a measure of the population. At the beginning of period 1, there are two players: an elite 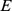 of 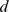-measure 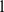, who appropriates oil and gas (the resources), and a civilian 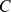 of 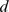-measure 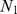, who has labor force. Resources can be either exported or sold domestically, while labor activity can be either productive or insurgent. The elites represent less than 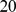
% of the total population (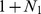), as shown in Assumption 1

#### Assumption 1



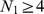



#### Remark 1

This parametric assumption, the so-called 80–20 rule, is consistent with the World Bank statistics that the richest 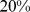 hold close to 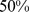 of the national income in most developing countries (Source: http://data.worldbank.org/indicator/SI.DST.05TH.20/countries).

The political regime is autocratic, and the elites rule the government. Revolution is broadly defined as any insurgent action or threat against the established political system. We do not distinguish rebellion, which is the attempt to revolt, from revolution, which is a successful rebellion. Accordingly, repression is defined as any counter-insurgency efforts of the elites.

### Extraction and Production

Oil and gas are “point resources” that are fixed in location and thus require sophisticated infrastructures to access, control, and transport. Only the elite can put together the necessary technology for exploration, production, and distribution, with the help of multinational oil and gas companies. The behavior of the multinationals are not modelled in the scope of this paper. According to latest Global Trade Analysis Project database statistics [19], labor in oil and gas extraction constitutes less than 2% of the total labor inputs, or less than 10% of the total inputs into oil and gas extraction in most of the developing world. For simplicity, it is assumed that resource extraction does not require labor input.

As is pre-contracted with the multinationals, in each period, the elite extracts one unit of resources and exports 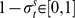 of resources at price 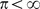, which is exogenously given and constant. The remaining 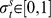 is sold domestically at price 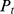, which is determined by a monopolistic mechanism to be discussed shortly. In total, the elite receives the period resource windfall of
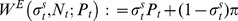
(1)


On the other hand, in each period, the civilian is endowed with one unit of time. The civilian purchases 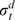 unit of resources from the elite in the form of energy and industrial infrastructure and supplies 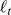 unit of labor to produce
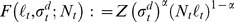
where 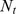 is the size of civilian population in period 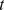, 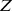 is the total factor productivity, and 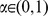 is the output elasticity of resources. Altogether, the civilian has the period net income of
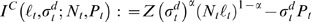
(2)


### Resource Market Equilibrium

To ensure that the economy has a comparative advantage in exporting, the following condition is assumed:

#### Assumption 2



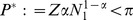



#### Remark 2

The following Equation (3) classifies that 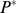 is the marginal return on resources when domestic production is at full capacity. If the world price is below 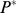, then all resources are consumed domestically.

Given a domestic resource price of 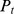, the civilians optimal choice is to equalize marginal product and cost of resources as
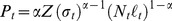
(3)Here, we have used the market clearing condition



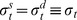



The elite moves simultaneously with the civilian, and can exert monopoly power only to maximize current time profit but not to maximize total survival time profits. Therefore, it is the elites optimal choice to equalize the marginal profits of export and domestic sales, as follows:
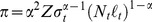
(4)


Altogether, the resource market equilibrium is
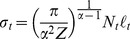
(5)

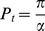
(6)


#### Remark 3

Assumption 2 ensures that the equality (5) is attainable.

By substitution, the elites period windfall is:
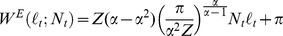



and the civilians net income is



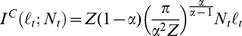



We can now concentrate on the political dynamics between the elite and the civilian.

### Revolution and Repression

At the beginning of period 1, the political statuses of the elites and the civilians are exogenously given. Over the course of the period, civilians can stage a rebellion using their non-productive time 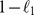. In response, the elites can defend themselves by directing 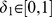 of the resource windfall to the counter-insurgency expenditure, such as mobilizing military forces, bribing coup leaders, and seeking external intervention. The probability that the elites retain power in period is assumed to be determined by the function 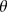, such that
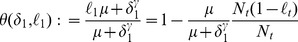
(7)Here, 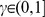 and 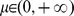 are parameters that represent the elites counter-insurgency effectiveness, which captures possible foreign military intervention. Since the early nineteenth century, Britain has played a key role in securing peace and prosperity in the Persian Gulf region. Following World War II, Britain scaled back its military presence around the world because of its economic problems. When Britain announced plans to withdraw troops from the Gulf region, the sheiks of the region asked the British to stay to ensure stability. For more information about oil-related and gas-related foreign intervention, see [20]. The restriction 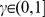 ensures that 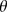 is concave in 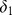. Given 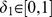, the rightmost term of Equation (7) is decreasing in 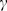. The spillovers of conflict into neighboring regions and the consequential countermeasures such as military intervention, economic sanctions and humanitarian aid are not explicitly considered in this paper.

The elites contest success function, i.e., Equation (7), is a fusion of two streams in the literature. The first presentation is similar to “gun choice,” as seen in [17]. The second presentation has the essence of probabilistic voting 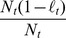, which follows [21]. Because probabilistic voting eliminates the impact of the size of civilian population on political change, we make it comparable by assuming that only 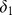, the elites counter-insurgency expenditure as a proportion of total resource windfall, plays a role in 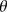.

#### Remark 4

By the law of large numbers, the situation in which the civilian revolts with some effort is equivalent to the real-world situation, in which some organized civilians fight against the elites with full effort headed by a coup leader, while the remaining civilians continue to work with full effort. Modeling collective action of civilians is complex [22] and is beyond the scope of this paper.

The following properties of 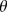 are in order. First, the elite retains power when there is no revolution:
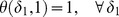



Second, the marginal regime-stabilization effect of the elites counter-insurgency efforts is positive and diminishing:
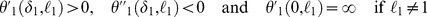


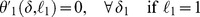



Third, the marginal regime-stabilization effect of the civilians productive commitment is positive and constant:
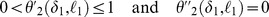



Last, the elites counter-insurgency efforts and the civilians productive commitment are substitutes:
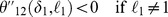



### Aftermath of Insurgency

Insurgency is rewarding but risky. If the insurgency is successful, then the elite dies, and some “lucky” civilian of 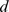-measure 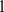 becomes the new elite. This leads to an expected loss of civilian population:
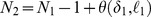
(8)


This indirectly affects productivity in period 2, and can be considered as the expected lethality of revolution. Additionally, violence always causes the civilian to forgo work earnings, no matter who wins.

#### Remark 5

Our model does not penalize the civilian if a rebellion is unsuccessful. Modeling this type of penalty requires a discrete function to capture the fact that the elite is penalized only if he or she revolts with an infinitely small effort and still fails, but not if he or she does not revolt. This treatment reduces the continuity and interior differentiability of the model, which are crucial to proving existence and uniqueness of the equilibrium. The expected loss of population already captures the dynamic trade-off of the civilian in relation to insurgency. Thus, we abstract the violence penalty to offer a theoretical model with a unique equilibrium solution that is econometrically identifiable.

### Strategic Interactions

Both the elite and the civilian have perfect information and move simultaneously in each period. Knowing the probabilistic regime switching and given the civilians labor supply 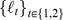, the elite chooses defence budget 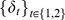 to obtain
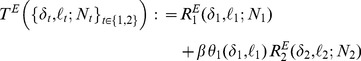
(9)where 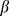 is the discount factor, and 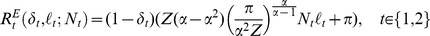
(10)is the elites period payoff net of counter-insurgency expenditures.

On the other hand, also knowing the probabilistic regime switching and given the elites defence budget 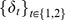, the civilian chooses labor supply 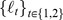 to obtain
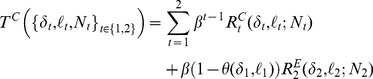
(11)where
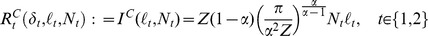
(12)is the civilian's period payoff.

#### Remark 6

Although it is more realistic to assume that both the elite and the civilian are risk-averse, this greatly complicates the math. However, letting the elite and the civilian be risk-neutral and assigning linear utilities to their period payoffs does not change the fundamental results of the model. In fact, it gives rise to an equilibrium with no labor supply and thus no domestic production, which better approximates a full-scale civil war.

### Equilibria of Resource-Related Conflict

Let 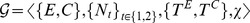 be the two-stage game, as defined in the previous section, where 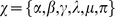 is the list of all model parameters. For the game 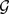, the following solution concept is adopted:

#### Definition 1

A sub-game perfect equilibrium is a pure-strategy profile 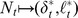 such that

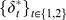
 maximizes 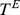, given 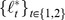,

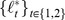
 maximizes 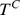, given 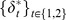,
- for any 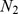 that is predetermined, 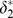 maximizes 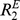, given 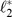,
- for any 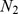 that is predetermined, 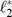 maximizes 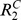, given 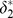.

Our first result of this paper now follows:

#### Theorem 1

*Let Assumptions 1 and 2 hold*.

1. *Let 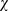 and 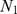 be fixed. There exists a unique sub-game perfect equilibrium to the game 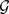, and it can be solved by backward induction*.
2. *Let 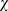 and 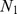 be fixed. The solution to the period-2 sub-game is 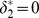 and 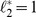, and the solution to the period-1 sub-game must be of one of the two forms below:*

- *peace with* 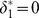 and 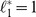, or
- conflict *with* 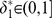 and 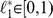

*When 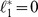, we call the equilibrium* civil war.

3. *Let other parameters in 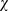 and 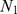 be fixed. There exists a 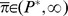 such that 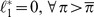. That is, the increase of world resource price will eventually lead to a civil war*.

*Proof. See* Appendix S1.

#### Remark 7

The equilibrium of repression and revolution discussed above offers a candidate mechanism for the resource-related conflict. As the elites take a large share of the domestic product, the civilians engage in insurgent activities to uplift their political status and raise the expected economic payoffs. The elites rely on resource windfall from exports and resort to outright repression, rather than economic reforms, to keep the civilians in check and to resolve their discontent. Interested readers can refer to, e.g., [23], for discussions on the effect of resource abundance on the political leaders behavior and political-regime determination. The subsequent escalation of attack and defence displaces labor and destabilizes the domestic environment, which adversely affects production. Consequently, resource exports emerge as the main source of national income, and power struggles over the control of resources prevail. Additionally, the incidence of social conflict parallels the economys increased dependence on resource exports, since higher world resource prices push up the cost of domestic production and intensify the civilians discontent. Because the equilibrium exists and is unique, it is possible to test it against real data. Indeed, our analytical result on the monotonicity of revolution (part 3 of the theorem) is consistent with the empirical finding of Besley and Persson [24], [25] that the oil export price is positively correlated with the incidence of civil war and that civil war is more prevalent among non-democratic oil producers.

### Consequences of Resource-Related Conflict

In this section, we investigate the (expected) Pareto inferiority of the equilibrium outcome of our model (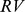 henceforth) as compared to two alternative models. The first-best alternative (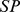 henceforth) has a social planner who solves the following program:
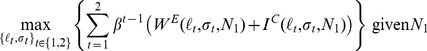
(13)


In the second-best alternative (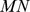 henceforth), the elite is an unchallengeable monarch:
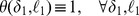



Clearly, the existence of social fractionalization and market monopoly in model 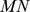 distinguishes it from 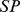, while the existence of conflict in 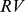 distinguishes it from 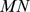.

We also compare our model to a resource-deficient economy (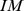 henceforth), in which there is no social fractionalization and all resources are imported. In the absence of monopoly, the representative civilian solves the following program:
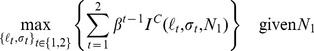
(14)


This 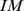 economy has a comparative disadvantage in resources but an advantage in market institutions.

Let 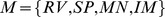 be the collection of models as discussed above. Accordingly, for 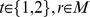, let 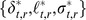 denote the equilibrium solutions, 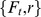 denote the equilibrium domestic production, and 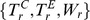 denote the equilibrium payoffs of the civilian, the elite, and the economy as a whole. When appropriate, we let 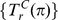 denote the civilians equilibrium payoff, given the world resource price 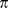. The following results are in order:

#### Theorem 2

Let Assumptions 1 and 2 hold, and let 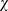 be fixed.

1. *The existence of social fractionalization, market monopoly, and civil conflict leads to the under-provision of labor and resources and thus depresses domestic production:*

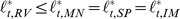


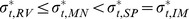


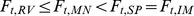



*As a result, resources and labor are displaced from the social optimum:*


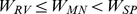


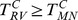

*The civilian is better off as a result of insurgency:* *Being challenged, however, the elite is worse off:*

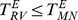

*All inequalities above are strict when conflict prevails, that is,* 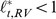.

2. *Let the conflict technology, i.e*, 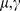, *be constant. If either* 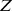, 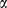 *or* 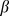 *is sufficiently small, then there exists a* 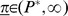, *such that*

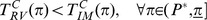



*In other words, civilians in a resource-abundant economy can be poorer than their counterparts in a resource-deficient economy with better market institutions.*

*Proof. See* Appendix S1.

#### Remark 8

The theorem above provides a potential explanation for the resource curse. First, domestic production and total social welfare are lower in an economy with resources that are appropriated by the elite rather than in an economy with resources that are allocated by a benevolent social planner. This captures the primary welfare loss, which is caused by market frictions and monopoly. Second, over the course of conflict, resources and labor are displaced from socially profitable activities. This captures the secondary welfare loss, which is caused by attack and defence. For these two reasons, resource-abundant countries may fail to become wealthy. Moreover, if climate conditions are unfavorable (i.e., 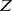 being small), if domestic production is labor-intensive (i.e., 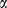 being small), or if the economy is trapped in a prolonged war (i.e., 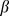 being small), then resource abundance is a curse. This is because the opportunity cost of conflict, as measured by the foregone growth potential, can be immense. Only a minority of civilians will become the new elite and reap the benefits, thus leaving the majority to handle the aftermath of conflict. In contrast, for a resource-deficient economy, domestic-oriented industrialization is a better choice for development. Indeed, according to the recent finding of Caselli and Tesei, resource-deficient countries are more likely to be democratic (Source: http://www.nber.org/papers/w17601). Thus, resource-rich countries may eventually be poorer than resource-poor countries.

## Results and Discussion

In this section, we report the numerical results of our mathematical model. We begin by calibrating the baseline to replicate stylized facts of the resource curse and civil conflict. We then conduct sensitivity analyses with parameters in the production function that are related to climate condition and resource dependence. We also investigate the policy implications of resource subsidy and the strategic aspect of domestic-resource sales.

### Baseline

For the baseline exercise, we assume 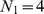, which means that civilians constitute 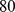
% and elites constitute 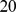
% of the total population. We set total factor productivity 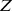 in the production function 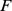 equal to 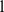. Having a developing country in mind, we set the parameter 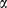 equal to 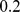, which is much lower than what is commonly used in the literature. The discount factor 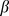, is set to 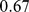, which implies a conflict length of roughly 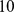 years, assuming an annual interest rate of 4%. The parameter 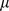 in the regime-switching function 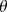 is set to 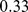, which implies that the elite is three times more likely to win when both the elite and the civilian battle at full strength. According to a recent research of Collier and Hoeffler, only 82 out of 336 rebellions in Africa from 1960 to 2001 were successful (Source: http://users.ox.ac.uk/ econpco/research/pdfs/MilitarySpendingandRisksCoups.pdf). The other parameter 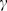 is set to 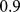, which implies that doubling 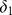, the elites counter-insurgency expenditure as a share of total resource windfall, roughly doubles the chance of retaining power when 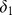 is within 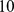
%, but chances are lower when it is greater than.

**Figure 2:**
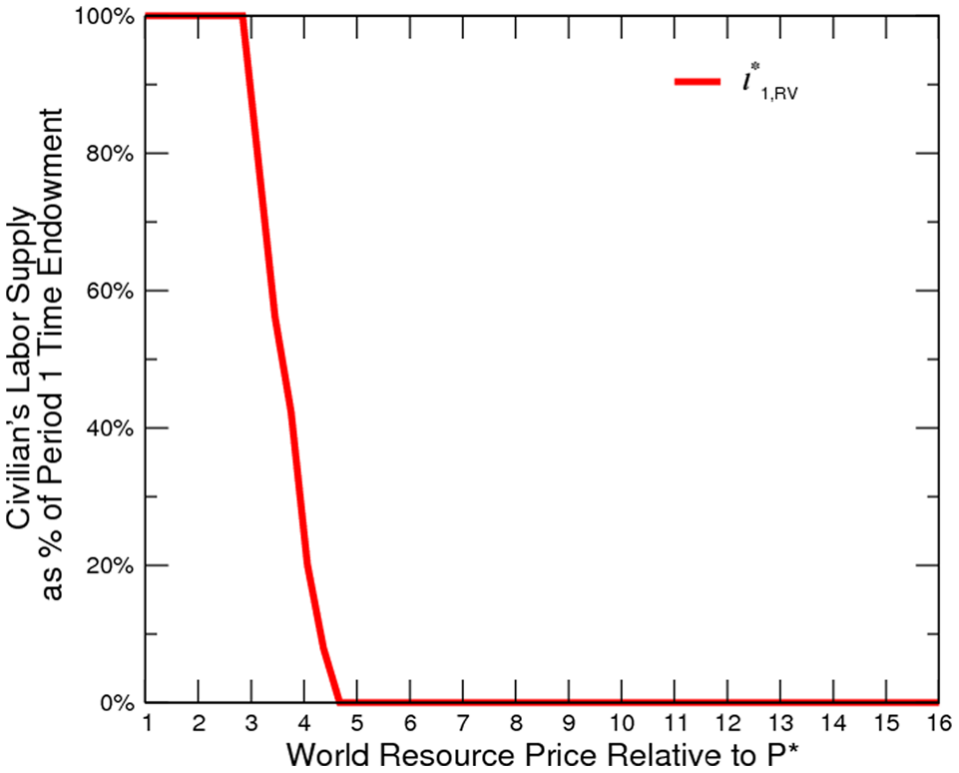
Civilan's Labor Supply at Various Price Levels.

**Figure 3:**
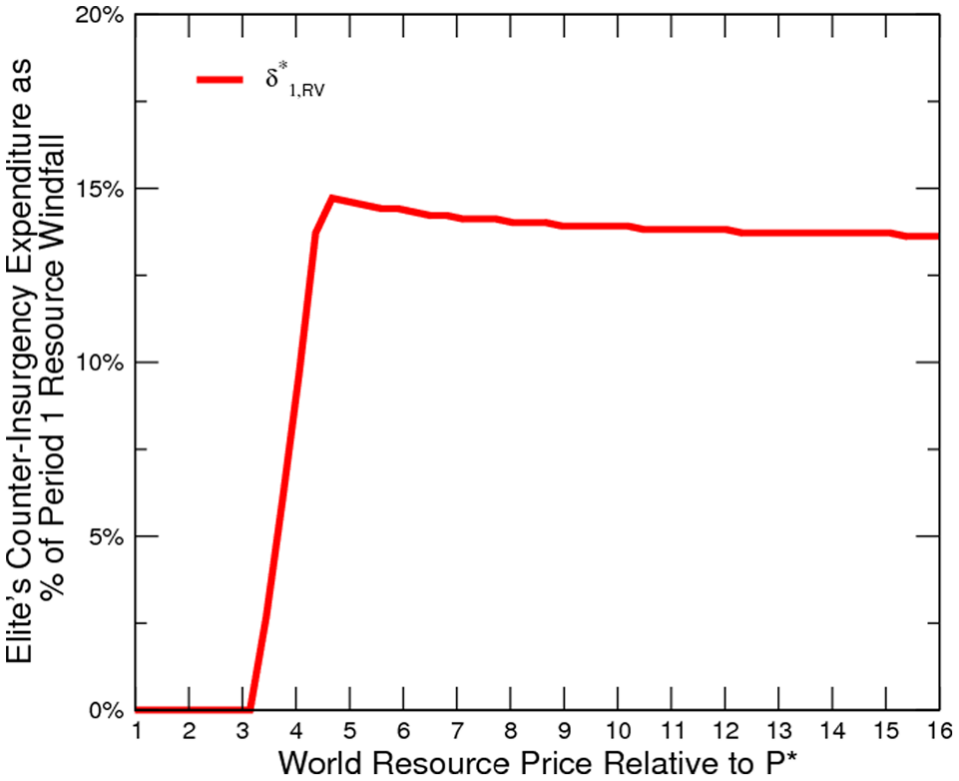
Elite's Counter-Insurgency Expenditure at Various Price Levels.

**Figure 4:**
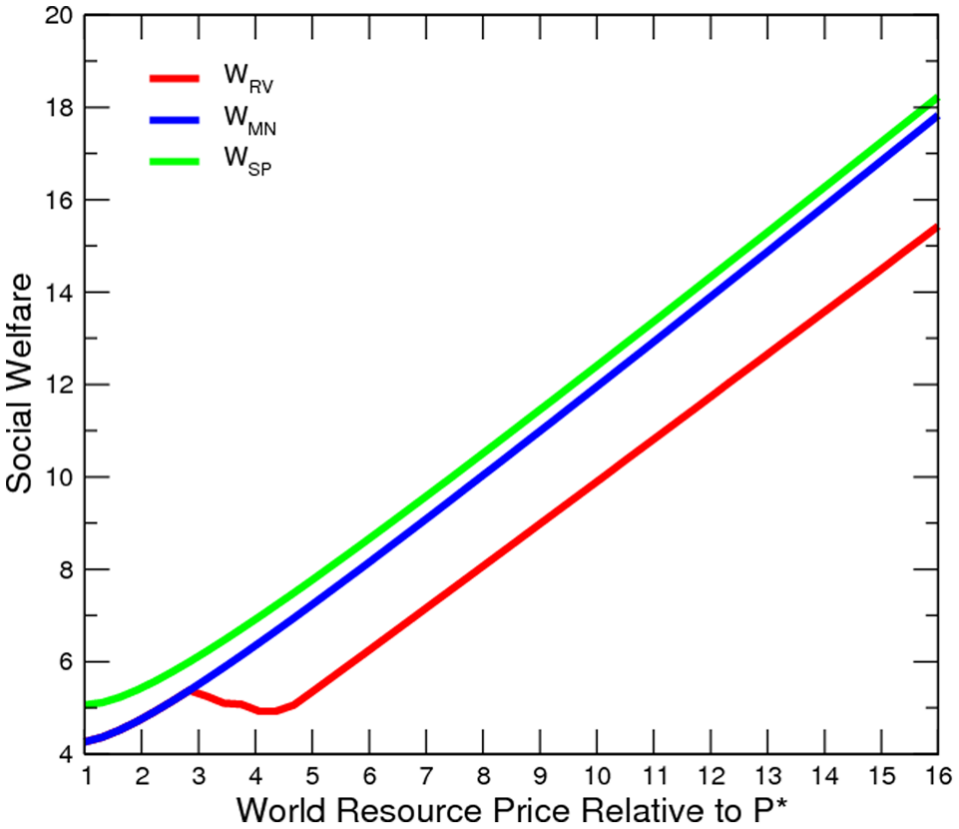
Social Welfare at Various Price Levels.

**Figure 5:**
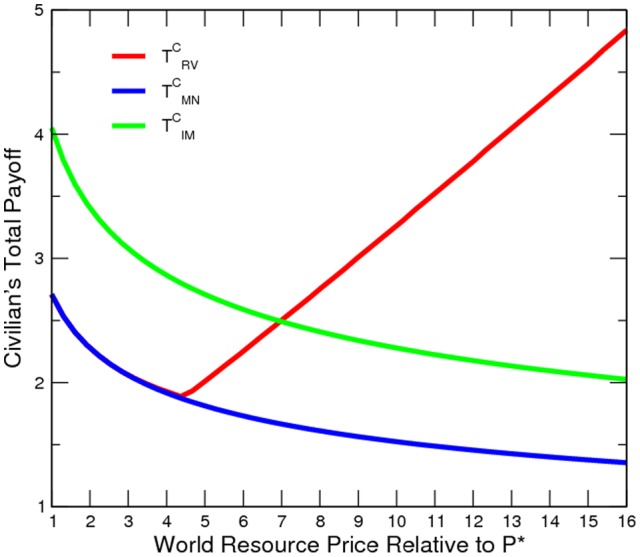
Civilan's Total Payoff at Various Price Levels.

The numerical results confirm our model prediction (Figures 2 to 5). Oil and gas prices are positively correlated to the incidence of conflict. Although conflict worsens social welfare, the civilian is in fact better off. An export boom (roughly a 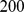
% price wedge) can relatively easily provoke resource-related civil conflict. However, a substantially higher world price (roughly a 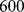
% price wedge) is required to compensate for the lost growth potential and to make civilians in a resource-abundant economy as successful as their resource-deficient counterparts, who have a more supportive market environment. This shows how easy it is to turn a resource fortune into a curse and how difficult it is to turn a curse into a fortune. In addition, the comparison across the four models, namely, 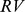, 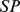, 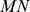 and 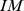, highlights the importance of governance reform in oil-and-gas-related conflict resolution and poverty reduction.

### Climate Change and the Incidence of Conflict

**Figure 6:**
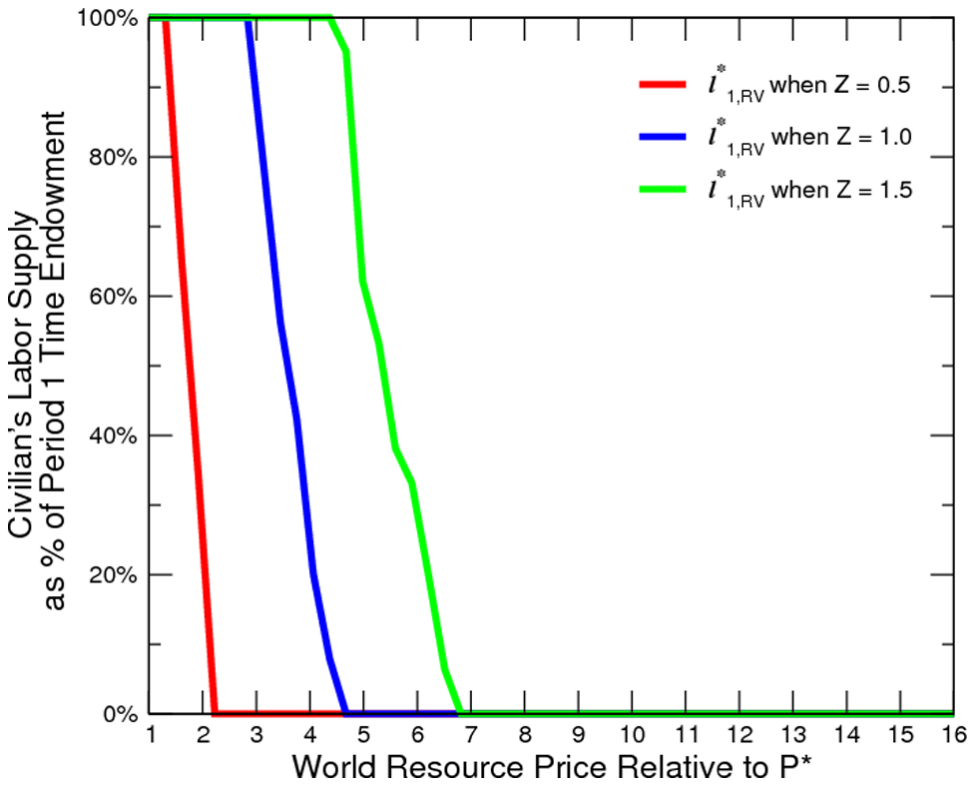
Climate Change and Incidence of Conflict.

In the context of domestic agricultural production, total factor productivity 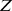 in the production function 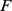 can be understood as climate conditions, such as rainfall variation. Our model can be applied to recent studies of global climate and civil conflict [26]–[28]. Intuitively, adverse climate conditions (i.e., 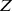 being small) lowers the return to legal labor activity, which causes the civilian to challenge the existing elites and to take over control of resources, and vice versa. This is confirmed in the simulation (Figure 6) where we alter the value of 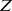 to 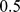 and 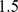, respectively. Other parameters stay the same as in the baseline. Our simulation also highlights the importance of productivity growth in oil-and-gas-related conflict resolution.

### Resource Dependence and the Incidence of Conflict

**Figure 7:**
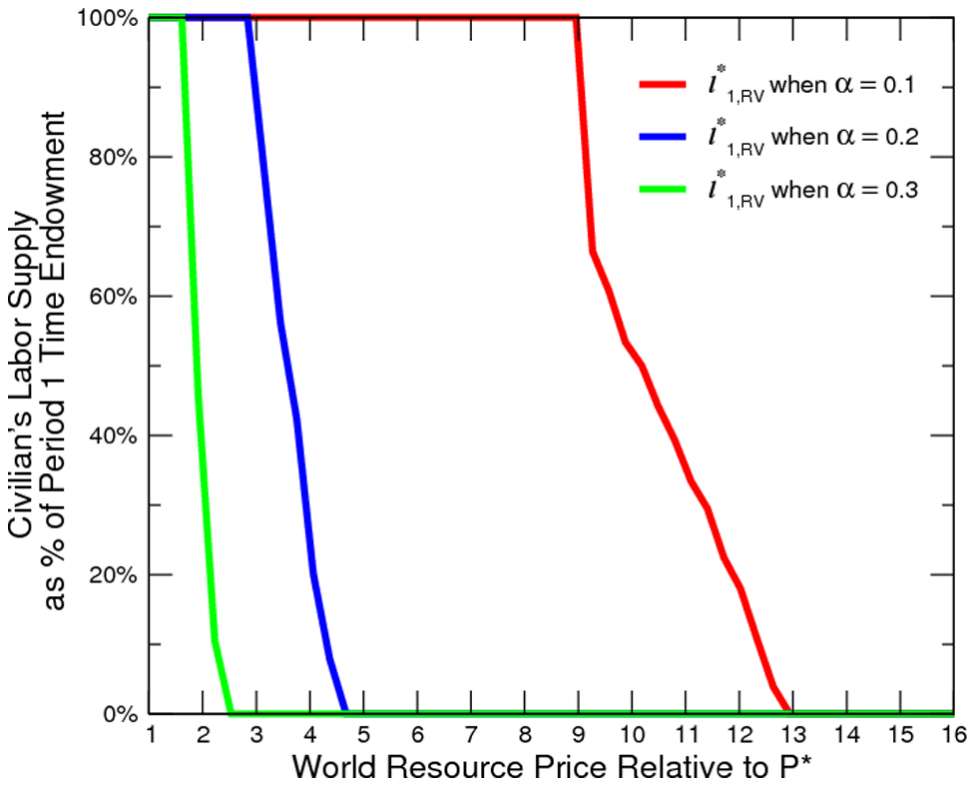
Resource Dependence and Incidence of Conflict.

By Euler's Theorem (see, e.g., [29]), the output elasticity of resources 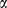 in the production function 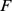 is also a measure of the economys dependence on resources. In the context of social fractionalization and monopolistic pricing, as 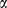 increases, the share of domestic output that is taken by the resource-owning elite increases, and thus the elite discontent increases. This is confirmed in the simulation (Figure 7) when we alter the value of 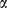 to 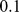 and 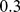, respectively. Other parameters stay the same as in the baseline. Intuitively, this suggests that, in an economy with less demand for oil and gas, high export prices are less likely to trigger conflict.

### Resource Subsidy

Our model predicts that a resource-rich country with weak governance will have higher prices of oil and gas and lower consumption in the domestic market. In fact, many oil-and-gas-rich countries have struggled with sub-par levels of energy consumption, as previously discussed. However, it is more often the case that oil-and-gas-rich countries have lower energy prices, even after accounting for transportation costs (Source: http://www.mytravelcost.com/petrol-prices/). This is counter-intuitive, as low resource prices should stimulate consumption.

To reconcile the observed reality, economic intuitions, and our model prediction, we postulate that domestic resources subside. We assume, ex post, that a fixed shared of the elites counter-insurgency fund is appropriated and spent as an ad valorem resource subsidy. In other words, the subsidies occur and become known to the civilian only after both 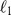 and 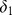 are determined. This redistribution scheme is never optimal, because it is arbitrary, conspired by the elite, and beyond the civilians strategic consideration. None of the fundamentals of our model is changed. Therefore, the same equilibrium would emerge even if domestic resource consumption were now subsidized. Social conflict may still linger and disrupt production. As a result, the resource consumption of a resource-abundant economy can be lower than that of a resource-deficient economy, despite a lower domestic price of resources.

**Figure 8:**
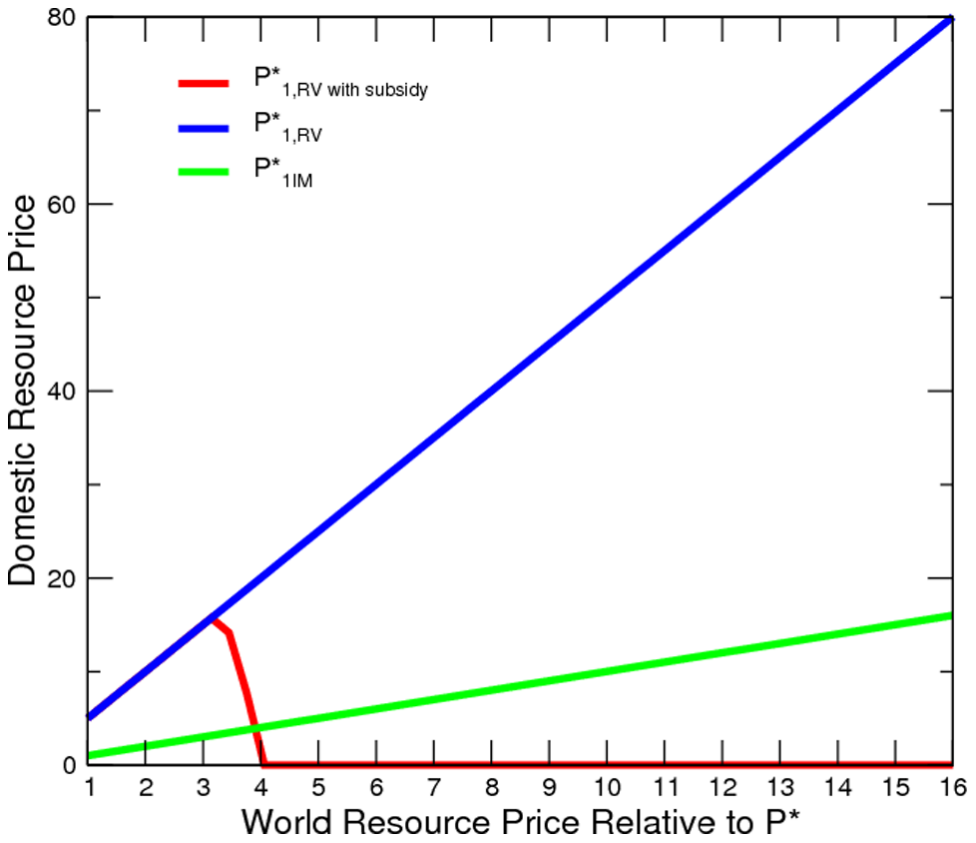
Domestic Resource Price at Various Price Levels.

**Figure 9:**
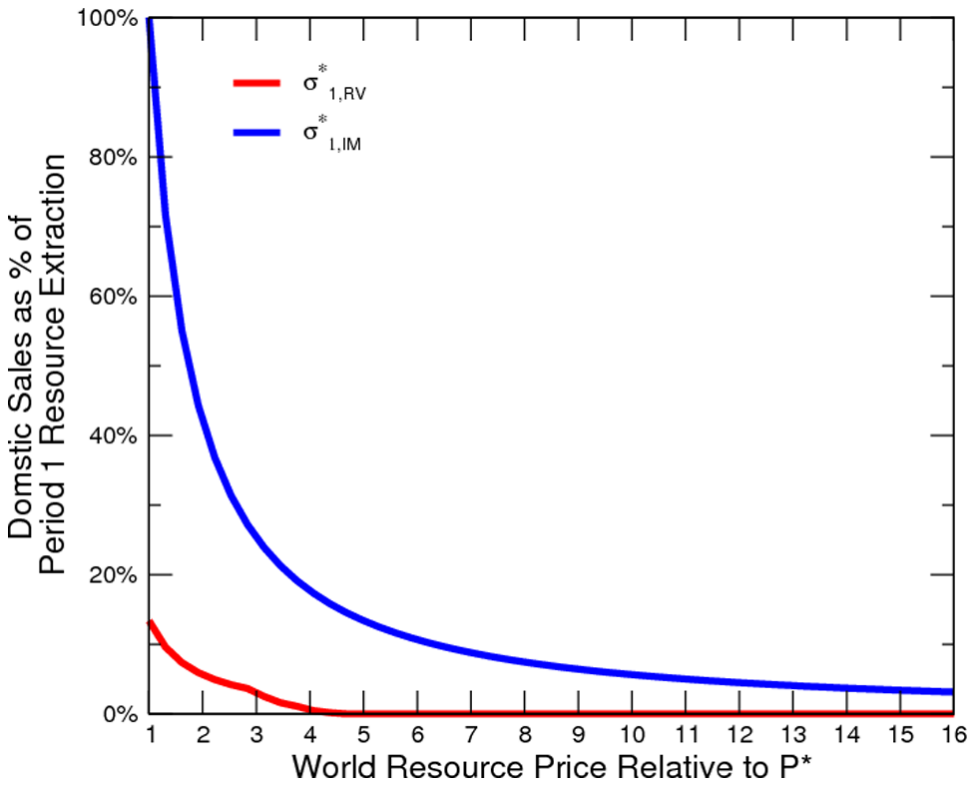
Domestic Resource Sales at Various Price Levels.

On top of the baseline, we assume that 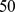
% of the counter-insurgency expenditure is directed to subsidize domestic resource consumption. Figures 8 and 9 show that subsidy is most likely to be superfluous. By assumption, the domestic price is zero when resource consumption is (more than) fully subsidized on a per-unit basis or when there is no demand. As long as the institution of transfer is not open to the involvement of the civilian, insurgency is still the default activity and domestic production ceases. Subsequently, the subsidization budget is squandered. This simulated result is a stronger demonstration of the model prediction of trade-pattern effects of conflict than that of Garfinkel *et al*. [17]. If subsidy is an ineffective stimulator, then domestic consumption of resources remains low. Civil conflict interrupts production, and thus resources are over-exported.

### Strategic Aspect of Resource Sales

Our model assumes that the elite moves simultaneously with the civilian in each period. Therefore, the elite can exert monopoly power only to maximize current time profit, rather than to maximize total survival time profits, because the choice of domestic resource sales has no effect on regime switching. This is a reasonable assumption, because many states with an elite class also have weak governance, and each individual elite controls a piece of the oil and gas resources. The consequential conflict between corrupt elites and frustrated civilians leads the economy into a development trap. The first-best solution is to transform the fractionalized state into a democratic nation and to streamline elite and civilian interests, as demonstrated in section with the social-planner model.

However, as a robustness check, we also consider a scenario in which the elites collude and form a strategic alliance to tip resource sales towards the domestic market. This decreases rebellion risk and maximizes the elites€ total survival time profit. In this alternative timing model, with other things being the same as in the baseline, the elite pre-commits a certain amount of resources to domestic use before the civilian commits to labor supply. The elite commits to a level of counter-insurgency expenditure. An extra step of backward induction is to optimize the elites strategic choice of resource sales, considering its effects on the elites later choice of counter-insurgency expenditure and the civilians simultaneous choice of labor supply. The existence and uniqueness of the equilibrium can be established by an approach that is similar to that in this paper, in which we use numerical simulation without rigorous mathematical analysis.

**Figure 10:**
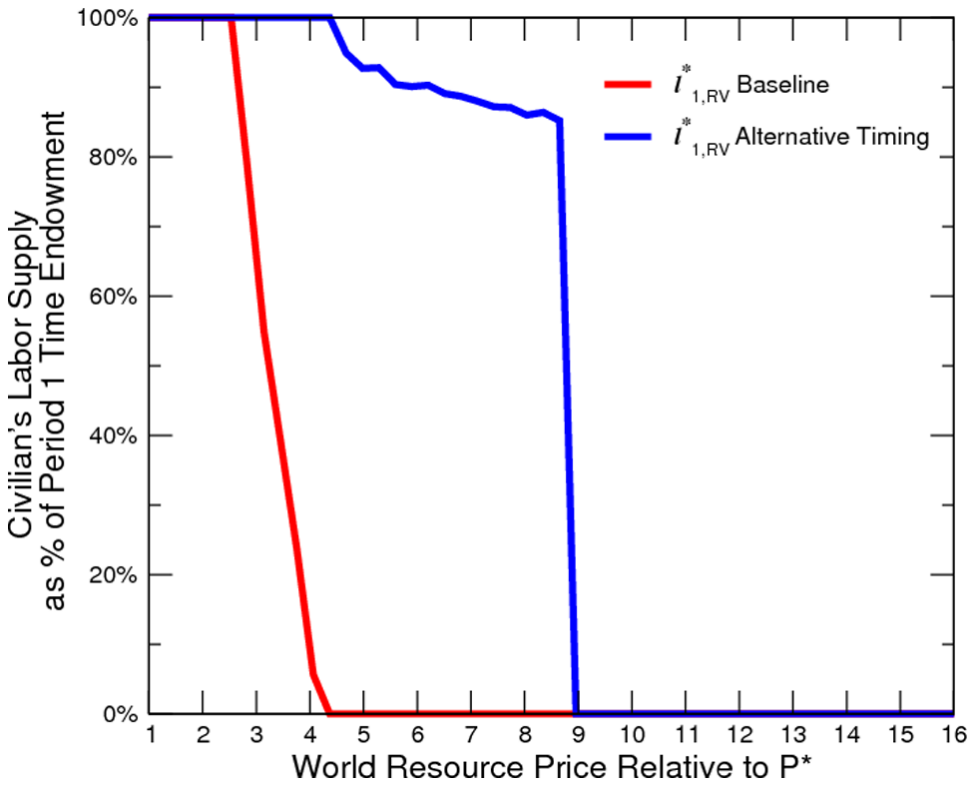
Civilian's Labor Supply under Alternative Timing.

**Figure 11:**
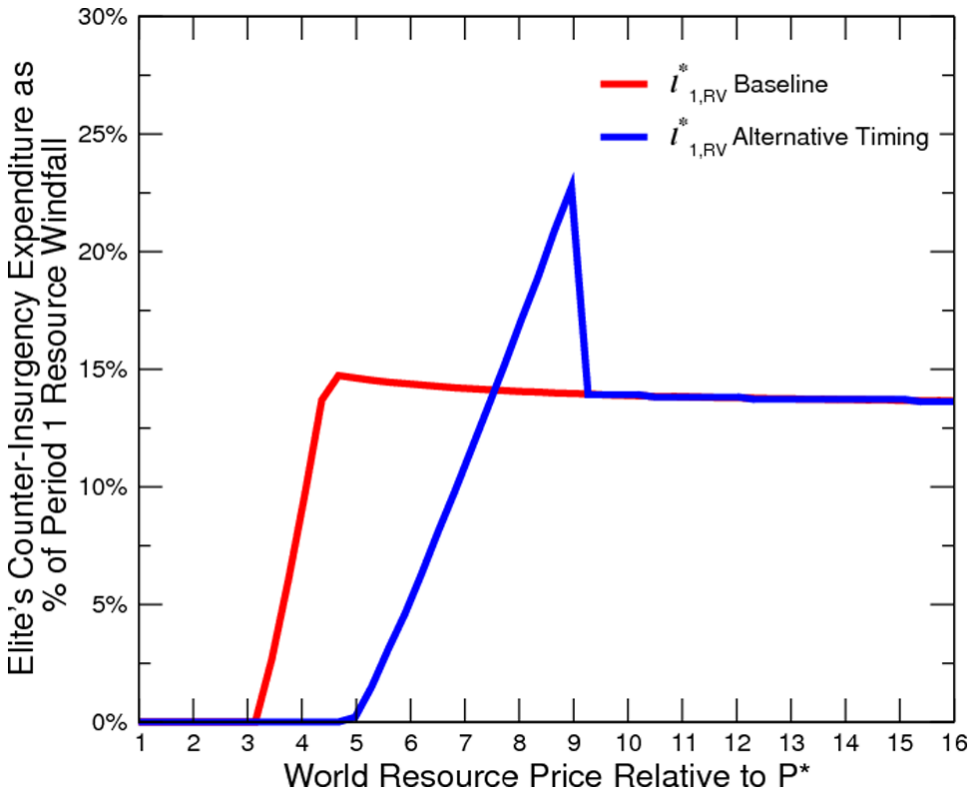
Elite's Counter-Insurgency Expenditure under Alternative Timing.

**Figure 12:**
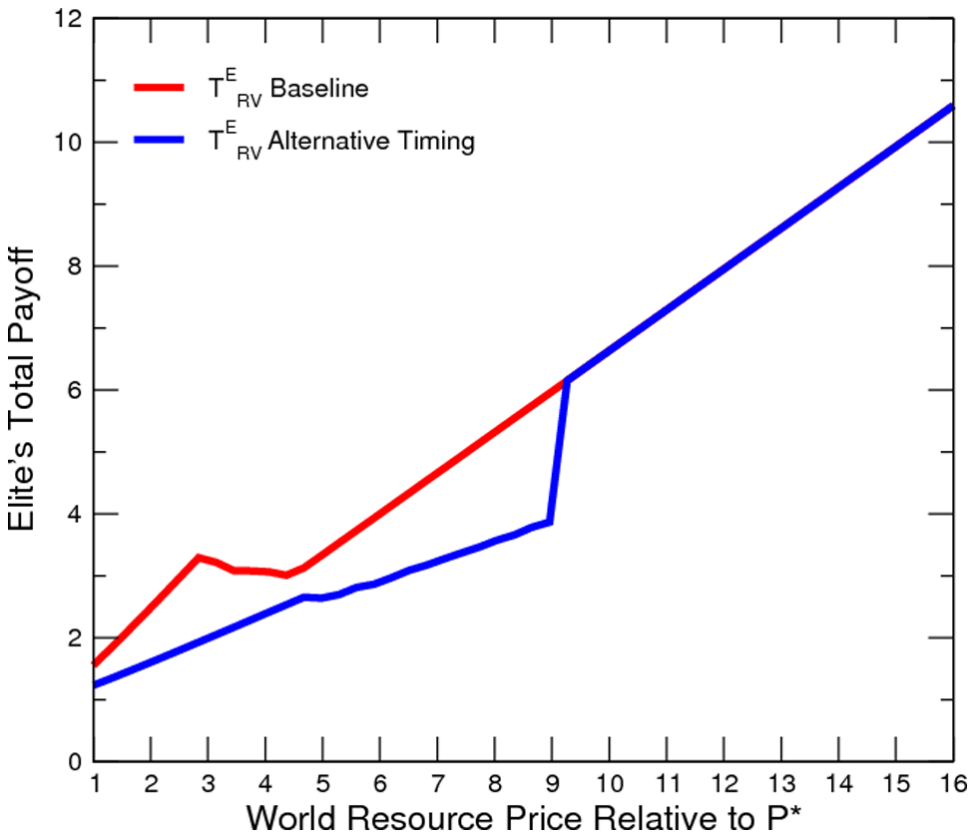
Elite's Total Payoff under Alternative Timing.

**Figure 13:**
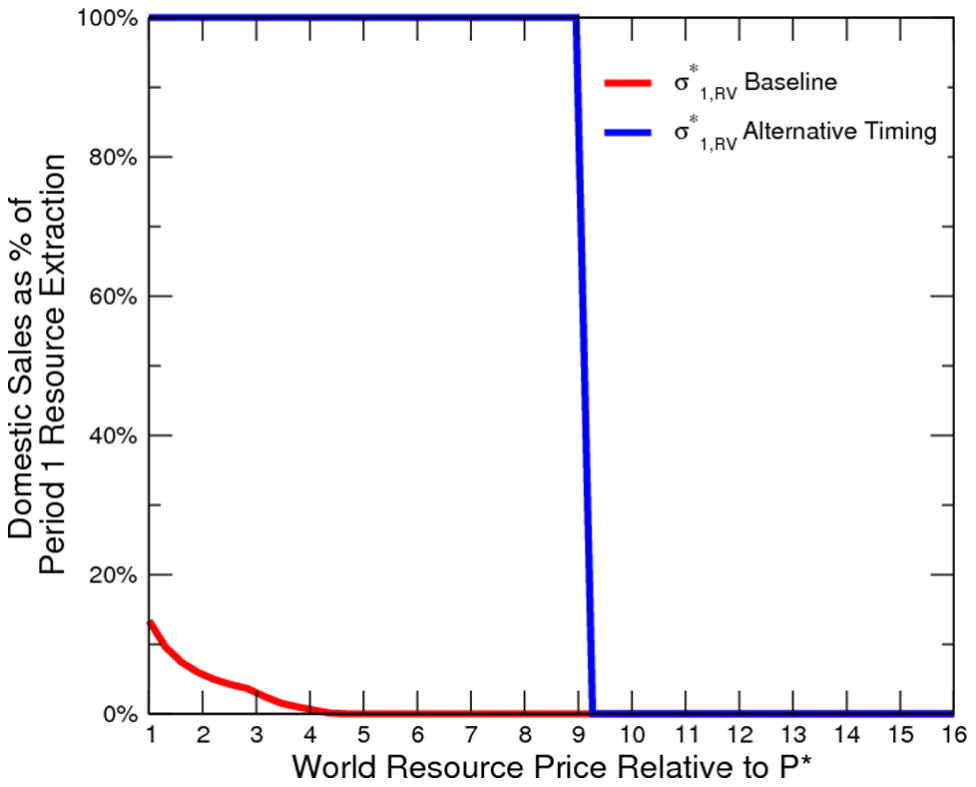
Domestic Resource Sales under Alternative Timing.

**Figure 14:**
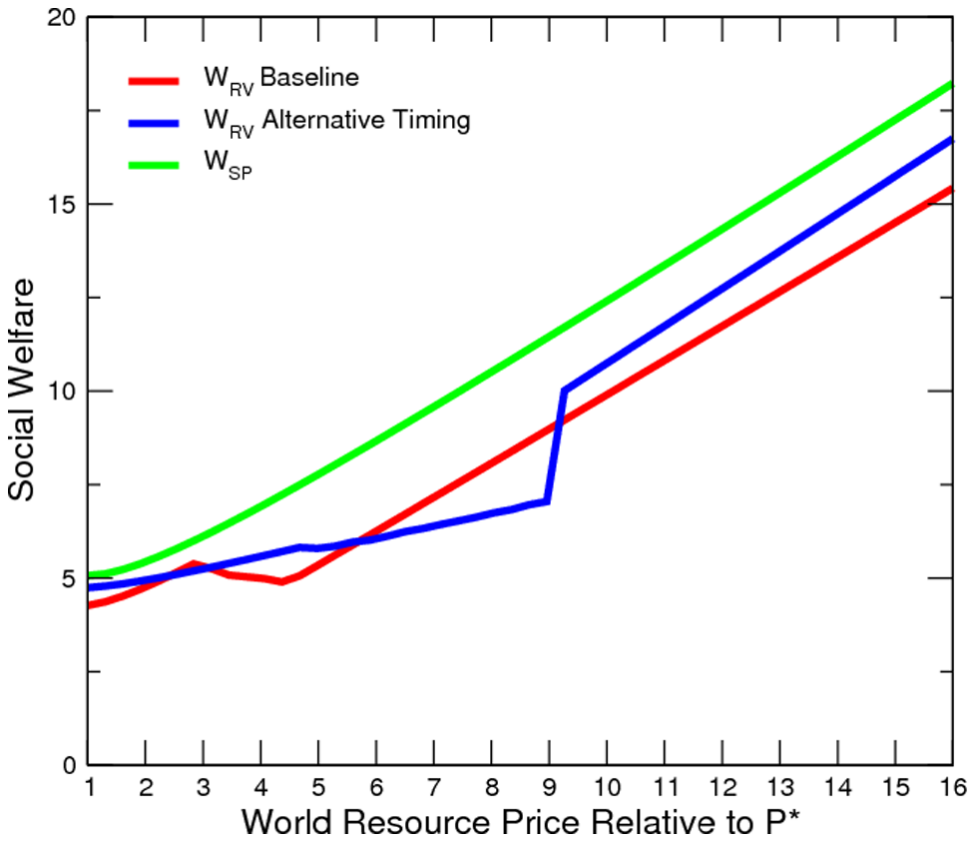
Social Welfare under Alternative Timing.

Our simulated results suggest that the economy is more peaceful in the alternative timing model (Figures 10 to 11). However, the elite is worse off than in the baseline (Figure 12). In other words, it is not profitable (from the elites point of view) or credible (from the elites point of view) for the elite to pre-commit domestic resource sales. Additionally, domestic resource sales are more volatile (Figure 13). When the world prices are low, the elite exports no resources; however, when world prices are sufficiently high, the elite exports all resources. Consequently, the welfare implication is mixed (Figure 14).

## Conclusions

Resource-abundant countries often show poor economic growth. This paper proposes a theoretical framework to study this paradox in the context of oil, gas, and conflict. The model addresses issues of social fractionalization, market friction, and civil conflict. It shows that the equilibrium between elite and civilian individual maximization behaviors can undermine the peaceful environment that is needed for industrialization and development. It can also further provoke resource-related conflict. The model predictions are consistent with observable facts.

The setup of the model is simple, and the parameter assumptions are non-stringent. Yet, it features a causality loop between resource dependence, conflict, and poor economic performance. Since the equilibrium of the model is proven to exist, and it is unique and eventually monotone, the model provides a well-defined structure for hypothesis testing and for the econometric identification of the mechanisms that underlie the resource curse.

## Supporting Information

Appendix S1 **Proofs of Theorem 1 and Theorem 2.**
(PDF)
