# Supplementary material for: Oil, Gas and Conflict: A Mathematical Model for the Resource Curse
Source: PLoS One. 2013 Jun 27;8(6):e66706. doi: 10.1371/journal.pone.0066706 (PMC3694977; doi:10.1371/journal.pone.0066706)
Supplement: Appendix S1 — Proofs of Theorem 1 and Theorem 2. (PDF) [file pone.0066706.s001.pdf]

**Appendix S1 for**
**Oil, Gas and Conflict: A Mathematical Model for the Resource Curse**

**Yiyong Cai[1,2,*], David Newth[1]**

**1 CSIRO Centre for Complex Systems Science, Commonwealth Scientific and Industrial Research Organisation, Canberra, ACT, Australia**
**2 Centre for Applied Macroeconomic Analysis, Australian National University, Canberra, ACT, Australia**
**∗ E-mail: Corresponding yiyong.cai@csiro.au**

Let us begin with some preliminary notation. Denote $\mathbb{R}_+ = [0, \infty)$. For any interval $[a, b] \subset \mathbb{R}_+$, we assume that it is endowed with the Euclidean metric and ordered by the usual order $\leq$. For any net $(x_\iota)_{\iota \in [a,b]}$, if $x_\iota = c$ for all $\iota$ with some constant $c$, then we shall identify the net with $c$. For any two real functions $f, g$ defined on the same domain $X$, we shall compactly denote the sum of $f, g$ such that $(f + g)(x) = f(x) + g(x)$. For a generic function $f : X \to \mathbb{R}_+$ such that $X = \times_{i=1}^{n}[a_i, b_i]$ for some $n \in \mathbb{N}$, $f'_i(x), f''_i(x), f''_{ij}(x)$ refer to the first-order, second-order, and cross (partial) derivatives at $x$, respectively, for any $x_i, x_j$ in the interior of $[a_i, b_i], [a_j, b_j]$. In the case when $x_i = a_i$ or $x_i = b_i$, we shall use the lower right and upper left first-order derivatives, respectively, for example,

$$f'_i(x) = f'_i(x)_+ := \lim_{\varepsilon \downarrow 0} \frac{f(x_i + \varepsilon, x_{-i}) - f(x_i, x_{-i})}{\varepsilon} \quad \text{for } x_i = a_i$$

and

$$f'_i(x) = f'_i(x)^- := \lim_{\varepsilon \downarrow 0} \frac{f(x_i - \varepsilon, x_{-i}) - f(x_i, x_{-i})}{-\varepsilon} \quad \text{for } x_i = b_i$$

where $x_{-i} = x \setminus \{x_i\}$.

## Proof of Theorem 1

Throughout this section we shall need the two Lemmata below:

**Lemma S-1.** *Let $f$ be a continuous function defined on the real interval $[a, b]$. If $f$ is differentiable on $(a, b)$, and $f'(c) > 0$ for all $c \in (a, b)$, then $f$ is increasing on $[a, b]$.*

*Proof.* Choose arbitrary $a \leq x < y \leq b$. By the Mean Value Theorem [1], there exists a $z$ such that $x < z < y$, and

$$f(y) - f(x) = f'(z)(y - x)$$

Given the assumption, $f'(z) > 0$ and hence $f(y) > f(x)$. □

**Lemma S-2.** *Let $f$ be a continuous function defined on the real interval $[a, b]$. If $f$ is twice differentiable on $(a, b)$, and in addition, $f''(z) \leq 0$ for all $z \in (a, b)$, then $f$ is also concave on $[a, b]$.*

*Proof.* By Theorem 6.4.6 of [1], $f$ is concave on $(a, b)$. Now fix $\varepsilon > 0$ and choose arbitrary $a \leq x < y \leq b$. For any $\lambda \in [0, 1]$, since $f$ is concave on $(a, b)$, we have

$$f(\lambda(x + \varepsilon) + (1 - \lambda)(y - \varepsilon)) \geq \lambda f(x + \varepsilon) + (1 - \lambda)f(y - \varepsilon)$$

Taking $\varepsilon \downarrow 0$ and by continuity of $f$,

$$f(\lambda x + (1 - \lambda)y) \geq \lambda f(x) + (1 - \lambda)f(y)$$

as to be proved. □

Now fix $\chi$ and $N_1$. We will proceed by backward induction and show the existence of at least one sub-game perfect equilibrium.

**Lemma S-3.** *The period-2 subgame equilibrium must satisfy $\delta_2^* = 0$ and $\ell_2^* = 1$. Given this, the elite and the civilian's total expected payoffs are*

$$T^E(\delta_1, \ell_1, N_1) = R_1^E(\delta_1, \ell_1, N_1) + \beta\, \theta(\delta_1, \ell_1) V_2^E(\delta_1, \ell_1, N_1)$$

*and*

$$T^C(\delta_1, \ell_1, N_1) = R_1^C(\delta_1, \ell_1, N_1) + \beta\big[(1-\theta(\delta_1, \ell_1))\, V_2^E(\delta_1, \ell_1, N_1) + V_2^C(\delta_1, \ell_1, N_1)\big]$$

*where*

$$V_2^E(\delta_1, \ell_1, N_1) = Z(\alpha - \alpha^2)\left(\frac{\pi}{\alpha^2 Z}\right)^{\frac{\alpha}{\alpha-1}} (N_1 - 1 + \theta(\delta_1, \ell_1)) + \pi \tag{S-1}$$

*and*

$$V_2^C(\delta_1, \ell_1, N_1) = Z(1 - \alpha)\left(\frac{\pi}{\alpha^2 Z}\right)^{\frac{\alpha}{\alpha-1}} (N_1 - 1 + \theta(\delta_1, \ell_1)) \tag{S-2}$$

*are the continuation values (i.e., equilibrium payoffs) of being the period-2 elite and civilian given $(\delta_1, \ell_1, N_1)$, respectively.*

*Proof.* Since the game terminates at the end of Period 2, there is no return to insurgency and counter-insurgency, which implies the first part of the lemma. The second follows readily by substitution. □

**Proposition S-1.** *A sub-game perfect equilibrium must be of one of the two forms below:*

- peace *with $\delta_1^* = 0$ and $\ell_1^* = 1$, or*
- conflict *with $\delta_1^* \in (0, 1]$ and $\ell_1^* \in [0, 1)$*

*Proof.* Suppose that a sub-game perfect equilibrium exists. Let $\{\delta_1^*, \ell_1^*\}$ be the equilibrium solutions. If $\ell_1^* = 1$, then there is no probabilistic regime-switching, and thus it must be $\delta_1^* = 0$.

Alternatively, if $\ell_1^* < 1$, we claim that $\delta_1^* > 0$. Suppose for a contradiction that $\ell_1^* < 1$ but $\delta_1^* = 0$. Choose $\varepsilon$ such that $0 < \varepsilon < 1$. By optimality,

$$\begin{aligned} & \frac{T^E(\varepsilon, \ell_1^*, N_1) - T^E(0, \ell_1 *, N_1)}{\varepsilon} \\ = \;& \beta\left(B + A\,\frac{\theta^2(\varepsilon, \ell_1^*) - \theta^2(0, \ell_1^*)}{\theta(\varepsilon, \ell_1^*) - \theta(0, \ell_1^*)}\right)\frac{\theta(\varepsilon, \ell_1^*) - \theta(0, \ell_1^*)}{\varepsilon} - AN_1\ell_1^* - \pi \\ \leq \;& 0 \end{aligned}$$

where

$$A = Z\alpha(1 - \alpha)\left(\frac{\pi}{\alpha^2 Z}\right)^{\frac{\alpha}{\alpha-1}} > 0$$

$$B = \pi + A(N_1 - 1) \geq 2A > 0$$

Taking $\varepsilon \downarrow 0$, we have

$$\Big(B + 2A\,\theta(0, \ell_1^*)\Big)\theta_1'(0, \ell_1^*) = \infty \leq \pi + AN_1\ell_1^*$$

which is impossible. □

**Lemma S-4** (Karush-Kuhn-Tucker Condition 1)**.** *Suppose that there exists a sub-game perfect equilibrium. Let* $\{\hat{\delta_1}, \hat{\ell_1}\}$ *be the set of equilibrium solutions. Define the function* $\Delta$ *as below*

$$\Delta(\delta_1, \ell_1) = \Big(B + 2A\theta(\delta_1, \ell_1)\Big)\theta_1'(\delta_1, \ell_1) - AN_1\ell_1 \tag{S-3}$$

*where* $A, B$ *are as in Proposition S-1. If* $\hat{\ell_1} < 1$*, then*

$$\Delta(\hat{\delta_1}, \hat{\ell_1}) \geq \pi$$

*If in addition,* $\hat{\delta_1} < 1$*, then*

$$\Delta(\hat{\delta_1}, \hat{\ell_1}) = \pi$$

*Proof.* Fix $0 \leq \hat{\ell_1} < 1$. By Proposition S-1, it follows that $\hat{\delta_1} > 0$. Choose $\varepsilon$ such that $0 < \varepsilon < \hat{\delta_1}$. By optimality,

$$\begin{aligned}
& \frac{T^E(\hat{\delta_1} - \varepsilon, \hat{\ell_1}, N_1) - T^E(\hat{\delta_1}, \hat{\ell_1}, N_1)}{-\varepsilon} + AN_1\hat{\ell_1} + \pi \\
= & \left(B + A\frac{\theta^2(\hat{\delta_1} - \varepsilon, \hat{\ell_1}, N_1) - \theta^2(\hat{\delta_1}, \hat{\ell_1}, N_1)}{\theta(\hat{\delta_1} - \varepsilon, \hat{\ell_1}, N_1) - \theta(\hat{\delta_1}, \hat{\ell_1}, N_1)}\right)\frac{\theta(\hat{\delta_1} - \varepsilon, \hat{\ell_1}, N_1) - \theta(\hat{\delta_1}, \hat{\ell_1}, N_1)}{-\varepsilon} \\
\geq & \ AN_1\hat{\ell_1} + \pi
\end{aligned}$$

Taking $\varepsilon \downarrow 0$, we have

$$\Big(B + 2A\theta(\delta_1, \ell_1)\Big)\theta_1'(\delta_1, \ell_1) \geq AN_1\hat{\ell_1} + \pi$$

as required by the first part of the lemma.

Suppose $\hat{\delta_1} < 1$. Choose $\varepsilon$ such that $0 < \varepsilon < 1 - \hat{\delta_1}$. By optimality,

$$\begin{aligned}
& \frac{T^E(\hat{\delta_1} + \varepsilon, \hat{\ell_1}, N_1) - T^E(\hat{\delta_1}, \hat{\ell_1}, N_1)}{\varepsilon} + AN_1\hat{\ell_1} + \pi \\
= & \left(B + A\frac{\theta^2(\hat{\delta_1} + \varepsilon, \hat{\ell_1}, N_1) - \theta^2(\hat{\delta_1}, \hat{\ell_1}, N_1)}{\theta(\hat{\delta_1} + \varepsilon, \hat{\ell_1}, N_1) - \theta(\hat{\delta_1}, \hat{\ell_1}, N_1)}\right)\frac{\theta(\hat{\delta_1} + \varepsilon, \hat{\ell_1}, N_1) - \theta(\hat{\delta_1}, \hat{\ell_1}, N_1)}{\varepsilon} \\
\leq & \ AN_1\hat{\ell_1} + \pi
\end{aligned}$$

Taking $\varepsilon \downarrow 0$, we have

$$\Big(B + 2A\theta(\delta_1, \ell_1)\Big)\theta_1'(\delta_1, \ell_1) \geq AN_1\hat{\ell_1} + \pi$$

which implies the second part of the lemma.

□

**Lemma S-5.** *For* $0 < \ell_1 < 1$*, the function* $\Delta$ *defined by (S-3) is strictly decreasing in both* $\delta_1$ *and* $\ell_1$*.*

*Proof.* First note that $\Delta$ is continuous on $[0,1]^2$ and differentiable on $(0,1)^2$ by construction. For the first argument, fix $\ell_1$ and choose $0 \leq \delta_a < \delta_b \leq 1$. By Lemma S-1, it suffices to show that $\Delta_1'(\delta_1, \ell_1) < 0$, $\forall \delta_1 \in (0,1)$, and we have

$$\begin{aligned}
\Delta_1'(\delta_1, \ell_1) &= B\theta_1''(\delta_1, \ell_1) + 2A\Big(\theta(\delta_1, \ell_1)\theta_1''(\delta_1, \ell_1) + \big(\theta_1'(\delta_1, \ell_1)\big)^2\Big) \\
&< 2A\Big(\theta_1''(\delta_1, \ell_1) + \big(\theta_1'(\delta_1, \ell_1)\big)^2\Big)
\end{aligned}$$

and

$$
\begin{aligned}
& \theta_1''(\delta_1,\ell_1)+\big(\theta_1'(\delta_1,\ell_1)\big)^2 \\
= & -\frac{\mu\gamma(1-\gamma)\delta_1^{\gamma-2}}{(\mu+\delta_1^{\gamma})^2}-2\big(\frac{\mu\gamma\delta_1^{\gamma-1}-}{(\mu+\delta_1^{\gamma})^2}\big)^2-2\frac{\mu\gamma^2\delta_1^{3\gamma-2}}{(\mu+\delta_1^{\gamma})^4}+(1-\ell_1)^2\big(\frac{\mu\gamma\delta_1^{\gamma-1}}{(\mu+\delta_1^{\gamma})^2}\big)^2 \\
< & \ 0
\end{aligned}
$$

as required.

For the second argument, fix $\delta_1$. Again it suffices to show that $\Delta_2'(\delta_1,\ell_1)<0$, $\forall \ell_1\in(0,1)$, and we have

$$
\begin{aligned}
\Delta_2'(\delta_1,\ell_1) &= B\theta_{12}''(\delta_1,\ell_1)+2A\Big(\theta(\delta_1,\ell_1)\theta_{12}''(\delta_1,\ell_1)+\theta_1'(\delta_1,\ell_1)\theta_2'(\delta_1,\ell_1)\Big)-AN_1 \\
&< 2A\Big(\theta_{12}''(\delta_1,\ell_1)+\theta_1'(\delta_1,\ell_1)\theta_2'(\delta_1,\ell_1)\Big)
\end{aligned}
$$

and

$$
\begin{aligned}
& \theta_{12}''(\delta_1,\ell_1)+\theta_1'(\delta_1,\ell_1)\theta_2'(\delta_1,\ell_1) \\
= & -\frac{\mu\gamma\delta_1^{\gamma-1}}{(\mu+\delta_1^{\gamma})^2}+(1-\ell_1)\frac{\mu\gamma\delta_1^{\gamma-1}}{(\mu+\delta_1^{\gamma})^2} \\
< & \ 0
\end{aligned}
$$

as required. □

**Lemma S-6** (Karush-Kuhn-Tucker Condition 2)**.** *Suppose that there exists a sub-game perfect equilibrium. Let $\{\hat{\delta_1},\hat{\ell_1}\}$ be the set of equilibrium solutions. Define the function $\Lambda$ as below*

$$
\Lambda(\delta_1,\ell_1)=\frac{N_1}{\beta\,\theta_2'(\delta_1,\ell_1)}-2\alpha\theta(\delta_1,\ell_1)-\frac{V}{U}>0 \tag{S-4}
$$

*where*

$$
U=Z(1-\alpha)\left(\frac{\pi}{\alpha^2 Z}\right)^{\frac{\alpha}{\alpha-1}}>0
$$
$$
V=U-\alpha U\,(N_1-2)
$$

*If $\hat{\ell_1}=1$, then*

$$
\Lambda(\hat{\delta_1},\hat{\ell_1})\geq \pi/U
$$

*If $\hat{\ell_1}=0$, then*

$$
\Lambda(\hat{\delta_1},\hat{\ell_1})\leq \pi/U
$$

*If $0<\hat{\ell_1}<1$, then*

$$
\Lambda(\hat{\delta_1},\hat{\ell_1})=\pi/U
$$

*Proof.* We only show the proof for $0<\hat{\ell_1}<1$. The proofs for the other two cases follow the same line of reasoning.

Fix $0<\hat{\ell_1}<1$. Choose $\varepsilon$ such that $0<\varepsilon<1-\hat{\ell_1}$. By optimality,

$$
\begin{aligned}
& \frac{T^C(\hat{\delta_1},\hat{\ell_1}+\varepsilon,N_1)-T^C(\hat{\delta_1},\hat{\ell_1},N_1)}{\varepsilon} \\
= & \ UN_1-\Big(\alpha U\frac{\theta^2(\hat{\delta_1},\hat{\ell_1}+\varepsilon)-\theta^2(\hat{\delta_1},\hat{\ell_1})}{\theta(\hat{\delta_1},\hat{\ell_1}+\varepsilon)-\theta(\hat{\delta_1},\hat{\ell_1})}+V+\pi\Big)\beta\,\frac{\theta(\hat{\delta_1},\hat{\ell_1}+\varepsilon)-\theta(\hat{\delta_1},\hat{\ell_1})}{\varepsilon} \\
\leq & \ 0
\end{aligned}
$$

Taking $\varepsilon \downarrow 0$, we have

$$UN_1 - \Big(2\alpha U\,\theta(\hat{\delta_1},\hat{\ell_1}) + V + \pi\Big)\beta\theta_2'(\hat{\delta_1},\hat{\ell_1}) \le 0 \tag{S-5}$$

By construction, $\theta_2'(\hat{\delta_1},\hat{\ell_1})$ is strictly positive, and hence the inequality (S-5) is equivalent to

$$\Lambda(\hat{\delta_1},\hat{\ell_1}) \le \pi/U \tag{S-6}$$

Now choose $\varepsilon$ such that $0 < \varepsilon < \hat{\ell_1}$. Again by optimality,

$$\begin{aligned}
&\frac{T^C(\hat{\delta_1},\hat{\ell_1}-\varepsilon,N_1) - T^C(\hat{\delta_1},\hat{\ell_1},N_1)}{-\varepsilon} \\
=\;& UN_1 - \Big(\alpha U\frac{\theta^2(\hat{\delta_1},\hat{\ell_1}-\varepsilon) - \theta^2(\hat{\delta_1},\hat{\ell_1})}{\theta(\hat{\delta_1},\hat{\ell_1}-\varepsilon) - \theta(\hat{\delta_1},\hat{\ell_1})} + V + \pi\Big)\beta\,\frac{\theta(\hat{\delta_1},\hat{\ell_1}-\varepsilon) - \theta(\hat{\delta_1},\hat{\ell_1})}{-\varepsilon} \\
\ge\;& 0
\end{aligned}$$

Taking $\varepsilon \downarrow 0$, we have

$$UN_1 - \Big(2\alpha U\,\theta(\hat{\delta_1},\hat{\ell_1}) + V + \pi\Big)\beta\theta_2'(\hat{\delta_1},\hat{\ell_1}) \ge 0 \tag{S-7}$$

which is equivalent to

$$\Lambda(\hat{\delta_1},\hat{\ell_1}) \ge \pi/U$$

as required. □

**Lemma S-7.** *The function $\Lambda$ defined by (S-4), is strictly increasing in $\delta_1$ and strictly decreasing in $\ell_1$.*

*Proof.* First note that $\Lambda$ is continuous on $[0,1]^2$ and differentiable on $(0,1)^2$ by construction. For the first argument, fix $\ell_1$ and choose $0 \le \delta_a < \delta_b \le 1$. By Lemma S-1, it suffices to show that $\Lambda_1'(\delta_1,\ell_1) > 0$, $\forall \delta_1 \in (0,1)$, and we have

$$\begin{aligned}
\Lambda_1'(\delta_1,\ell_1) &= -\frac{N_1}{\beta\left(\theta_2'(\delta_1,\ell_1)\right)^2}\theta_{12}''(\delta_1,\ell_1) - 2\alpha\theta_1'(\delta_1,\ell_1) \\
&= \frac{1}{\mu}\Big(\frac{N_1}{\beta} - 2\alpha(1-\ell_1)\big(\frac{\mu}{\mu+\sigma^\gamma}\big)^2\Big)\gamma\delta_1^{\gamma-1} \\
&> 0
\end{aligned}$$

as required.

For the second argument, fix $\delta_1$. Again it suffices to show that $\Lambda_2'(\delta_1,\ell_1) < 0$, $\forall \ell_1 \in (0,1)$, and we have

$$\begin{aligned}
\Lambda_2'(\delta_1,\ell_1) &= -\frac{N_1}{\beta\,(\theta_2'(\delta_1,\ell_1))^2}\theta_2''(\delta_1,\ell_1) - 2\alpha\theta_2'(\delta_1,\ell_1) \\
&= -2\alpha\theta_2'(\delta_1,\ell_1) \\
&< 0
\end{aligned}$$

as required.

□

**Lemma S-8.** *Holding $\ell_1$ constant, $T^E(\cdot,\ell_1)$ as defined in Lemma S-3 is concave in $\delta_1$; and holding $\delta_1$ constant, $T^C(\delta_1,\cdot)$ as defined in Lemma S-3 is concave in $\ell_1$.*

*Proof.* Fix $\ell_1$. For any $\delta_1 \in (0,1)$, note that by Lemma S-5,

$$T^E{}''_1(\delta_1, \ell_1) = \Delta'_1(\delta_1, \ell_1) < 0$$

It follows by Lemma S-2 that $T^E(\delta_1, \ell_1)$ is concave on $[0,1]$. This shows the first part of the lemma.

Now fix $\delta_1$. Note that $R_1^C(\delta_1, \cdot), \theta(\delta_1, \cdot), -\theta(\delta_1, \cdot)$ are affine in $\ell_1$, which implies that $-\theta^2(\delta_1, \cdot)$ is concave in $\ell_1$. As $T^C(\delta_1, \cdot)$ is the positive linear combination of $R_1^E(\delta_1, \cdot)$, $\theta(\delta_1, \cdot)$, $-\theta(\delta_1, \cdot)$ and $-\theta^2(\delta_1, \cdot)$, it is also concave in $\ell_1$. This proves the second part of the lemma. □

**Proposition S-2.** *There exists at least one sub-game perfect equilibrium, and it can be solved by backward induction.*

*Proof.* By construction $T^E$ and $T^C$ are continuous in $\delta_1$ and $\ell_1$, and the actions spaces, both being $[0,1]$, are nonempty compact convex subsets of $\mathbb{R}_+$. Together with Lemma S-8, the existence of a pure-strategy Nash equilibrium follows by Theorem 1.2 of [2]. By Proposition 172.1 of [3], this strategy profile derived by backward induction is also a sub-game perfect equilibrium. □

Given Propositions S-1 and S-2, it suffices to prove the uniqueness of the sub-game perfect equilibrium by showing that two distinct equilibria cannot coexist:

**Lemma S-9.** *A peace equilibrium cannot not coexist with a conflict equilibrium.*

*Proof.* Suppose that there are two coexisting equilibrium solutions $\{0, 1\}$ and $\{\hat{\delta_1}, \hat{\ell_1}\}$ such that $0 < \hat{\delta_1} \leq 1$ and $0 \leq \hat{\ell_1} < 1$. By Lemmata S-6 to S-7, we have a contradiction

$$\pi/U \leq \Lambda(0, 1) < \Lambda(0, \hat{\ell_1}) < \Lambda(\hat{\delta_1}, \hat{\ell_1}) \leq \pi/U$$

where $U$ is as in Lemma S-6. □

**Lemma S-10.** *Two distinct conflict equilibria cannot coexist with the same level of productive activities.*

*Proof.* Let $\{\tilde{\delta_1}, \tilde{\ell_1}\}$ and $\{\hat{\delta_1}, \hat{\ell_1}\}$ be the coexisting equilibrium solutions. Suppose that $\tilde{\ell_1} = \hat{\ell_1} \equiv \ddot{\ell_1} < 1$. Without loss of generality, let $0 \leq \hat{\delta_1} < \tilde{\delta_1} \leq 1$. By Lemmata S-4 to S-5, we have a contradiction

$$\pi = \Delta(\hat{\delta_1}, \ddot{\ell_1}) \leq \Delta(\tilde{\delta_1}, \ddot{\ell_1}) < \Delta(\hat{\delta_1}, \ddot{\ell_1}) \tag{S-8}$$

□

**Lemma S-11.** *Two distinct conflict equilibria cannot coexist.*

*Proof.* Let $\{\tilde{\delta_1}, \tilde{\ell_1}\}$ and $\{\hat{\delta_1}, \hat{\ell_1}\}$ be the coexisting equilibrium solutions. Given Lemma S-10, suppose, without loss of generality, that $0 \leq \hat{\ell_1} < \tilde{\ell_1} < 1$. If $0 < \hat{\delta_1} < \tilde{\delta_1} \leq 1$, then by Lemmata S-4 to S-5, we have a contradiction

$$\pi \leq \Delta(\tilde{\delta_1}, \tilde{\ell_1}) < \Delta(\hat{\delta_1}, \hat{\ell_1}) = \pi$$

If alternatively $0 \leq \tilde{\delta_1} \leq \hat{\delta_1} \leq 1$, then by Lemmata S-6 to S-7, we have another contradiction

$$\pi/U = \Lambda(\tilde{\delta_1}, \tilde{\ell_1}) < \Lambda(\hat{\delta_1}, \hat{\ell_1}) \leq \pi/U$$

where $U$ is as in Lemma S-6.

□

**Proposition S-3.** *The sub-game perfect equilibrium is unique.*

*Proof.* This follows readily by Lemmata S-9 to S-11. □

**Lemma S-12.** *Let other parameters in* $\chi$ *be fixed. For* $U, \Lambda$ *as defined in Lemma S-6, there exists a* $\overline{\pi} \in (P^*, \infty)$ *such that* $\Lambda < \pi/U$, $\forall \pi > \overline{\pi}$.

*Proof.* For any $\pi$, let $U(\pi)$ be the corresponding $U$ as defined in Lemma S-6. It is not difficult to see that $U$ is strictly decreasing in $\pi$. By Lemma S-7, we have

$$\Lambda(\delta_1, \ell_1) \leq \Lambda(1, 0) < \infty$$

Therefore, there exists a $\pi_a$ such that $\frac{\pi_a}{U(\pi_a)} = \Lambda(1, 0)$. Now letting $\overline{\pi} = \max\{\pi_a, P^*\}$ gives the result. □

We are now ready to complete the proof of Theorem 1.

*Theorem 1.* By Propositions S-2 to S-3, we have proved the first two results of the theorem. Part 3 of the theorem follows by Lemmata (S-6) and (S-12). □

## Proof of Theorem 2

Throughout this section, we fix $\chi$ and $N_1$. We solve the models one by one, and compare the equilibrium results as we go.

*The SP model*: Clearly, at the optimum labor is fully supplied,

$$\ell^*_{t,SP} \equiv 1 \tag{S-9}$$

and the social planner must be indifferent between domestic sales and exports of resources, i.e.,

$$Z\alpha(\sigma^*_{t,SP})^{\alpha-1} (N_1)^{1-\alpha} \equiv \pi$$

or equivalently

$$\sigma^*_{t,SP} \equiv \left(\frac{\pi}{\alpha Z}\right)^{\frac{1}{\alpha-1}} N_1$$

which implies the social optimal levels of domestic production and welfare

$$F_{t,SP} \equiv Z \left(\frac{\pi}{\alpha Z}\right)^{\frac{\alpha}{\alpha-1}} N_1 \tag{S-10}$$

$$W_{SP} = (1+\beta)\left(\pi + Z\frac{1-\alpha}{\alpha}\alpha^{\frac{1}{1-\alpha}}\left(\frac{\pi}{\alpha}\right)^{\frac{\alpha}{\alpha-1}} N_1\right) \tag{S-11}$$

*The MN model*: As the monarch is unchallengeable, attack and defence are vague efforts for the civilian and the elite, respectively. Hence,

$$\{\delta^*_{t,MN}, \ell^*_{t,MN}\} \equiv \{0, 1\} \tag{S-12}$$

However, domestic sales of resources are determined by the monopoly pricing,

$$Z\alpha^2(\sigma^*_{t,MN})^{\alpha-1} (N_1)^{1-\alpha} \equiv \pi$$

which leads to suboptimal level of domestic resource consumption

$$\sigma^*_{t,MN} \equiv \left(\frac{\pi}{\alpha^2 Z}\right)^{\frac{1}{\alpha-1}} N_1 < \sigma^*_{t,SP}$$

and thus depresses domestic production, i.e.,

$$F_{t,MN} = Z\left(\frac{\pi}{\alpha^2 Z}\right)^{\frac{\alpha}{\alpha-1}} N_1 < F_{t,MN} \tag{S-13}$$

With social fractionalization and market monopoly, the civilian's and the elite's equilibrium payoffs are

$$T_{MN}^E = (1+\beta)\left(\pi + Z\frac{1-\alpha}{\alpha}\alpha^{\frac{2}{1-\alpha}}\left(\frac{\pi}{Z}\right)^{\frac{\alpha}{\alpha-1}} N_1\right) \tag{S-14}$$

$$T_{MN}^C = (1+\beta)\left(Z\frac{1-\alpha}{\alpha^2}\alpha^{\frac{2}{1-\alpha}}\left(\frac{\pi}{Z}\right)^{\frac{\alpha}{\alpha-1}} N_1\right) \tag{S-15}$$

which implies the level of social welfare

$$W_{MN} = (1+\beta)\left(\pi + Z\frac{1-\alpha^2}{\alpha^2}\alpha^{\frac{2}{1-\alpha}}\left(\frac{\pi}{Z}\right)^{\frac{\alpha}{\alpha-1}} N_1\right) \tag{S-16}$$

We claim that social fractionalization and market monopoly result in a deadweight loss of social welfare:

**Proposition S-4.** $W_{MN} < W_{SP}$.

*Proof.* The statement of the lemma is equivalent to

$$g(\alpha) := \frac{1+\alpha}{\alpha}\alpha^{\frac{1}{1-\alpha}} < 1 \quad \text{for all } \alpha \in (0,1)$$

We claim that the function $g$ is decreasing on $[0,1]$. By Lemma S-1, it suffices to show that $g'(\alpha) < 0, \forall \alpha \in (0,1)$, and we have

$$\begin{aligned} g'(\alpha) &= g(\alpha)\Big(\frac{2}{1-\alpha^2} + \frac{\ln\alpha}{(1-\alpha)^2}\Big) \\ &= g(\alpha)\Big(\frac{2}{1-\alpha^2} - \frac{2\sum_{k=1}^{\infty}\frac{1}{2k-1}\left(\frac{1-\alpha}{1+\alpha}\right)^{2k-1}}{(1-\alpha)^2}\Big) \\ &< g(\alpha)\Big(\frac{2}{1-\alpha^2} - \frac{2\left(\frac{1-\alpha}{1+\alpha}\right)}{(1-\alpha)^2}\Big) \\ &= 0 \end{aligned}$$

where we have used the Maclaurin series expansion of $\ln\alpha$. Fix $0 < \alpha < 1$. There exists $0 < x < 1$ such that $\alpha = \frac{1-x}{1+x}$, or $\ln\alpha = \ln(1-x) - \ln(1+x)$. Taking the Maclaurin series expansions gives $\ln\alpha = -2\sum_{k=1}^{\infty}\frac{x^{2k-1}}{2k-1}$, and the substitution of $x = \frac{1-\alpha}{1+\alpha}$ gives the result as required. Therefore,

$$g(\alpha) < \lim_{x\to 0} g(x) = 1 \quad \text{for all } \alpha \in (0,1)$$

□

*The RV model*: When regime-switching is possible, engaging in revolution is a potentially profitable business, and thus

$$\ell_{1,RV}^* \leq 1 \tag{S-17}$$

$$\theta(\delta_1^*, \ell_1^*) \leq 1 \tag{S-18}$$

$$N_2 = N_1 - 1 + \theta(\delta_1^*, \ell_1^*) \leq N_1 \tag{S-19}$$

In response to the civilian's low level of productive activity, the elite exports more and commits less resources for domestic use, which shrinks production,

$$\sigma_{t,RV}^* = \left(\frac{\pi}{\alpha^2 Z}\right)^{\frac{1}{\alpha-1}} N_t \ell_{t,RV}^* \leq \sigma_{t,MN}^* \tag{S-20}$$

$$F_{t,RV} = Z\left(\frac{\pi}{\alpha^2 Z}\right)^{\frac{\alpha}{\alpha-1}} N_t \ell^*_{t,RV} \le F_{t,MN} \tag{S-21}$$

Because attack and defence are counter-productive, they lead to the loss of social welfare

$$\begin{aligned} W_{RV} &= (1-\delta^*_{1,RV} + \beta\theta(\delta^*_{1,RV}, \ell^*_{1,RV}))\pi \\ &+ \Big[\frac{1-\alpha^2-\delta^*_{1,RV}(\alpha-\alpha^2)}{\alpha^2} N_1\ell^*_{1,RV} + \beta\frac{1-\alpha^2}{\alpha^2}\big(N_1 - 1 + \theta(\delta^*_{1,RV}, \ell^*_{1,RV})\big)\Big] Z\alpha^{\frac{2}{1-\alpha}}\left(\frac{\pi}{Z}\right)^{\frac{\alpha}{\alpha-1}} \end{aligned} \tag{S-22}$$

It is not difficult to see that $W_{MN} \ge W_{RV}$.

Given the chance to lift their social status, the civilian is better off,

$$\begin{aligned} T^C_{RV} &= \beta\Big(1-\theta(\delta^*_{1,RV}, \ell^*_{1,RV})\Big)\pi \\ &+ \Big[\frac{N_1\ell^*_{1,RV}}{\alpha} + \beta\Big(1+\frac{1}{\alpha} - \theta(\delta^*_{1,RV}, \ell^*_{1,RV})\Big)\big(N_1 - 1 + \theta(\delta^*_{1,RV}, \ell^*_{1,RV})\big)\Big] Z(1-\alpha)\alpha^{\frac{1+\alpha}{1-\alpha}}\left(\frac{\pi}{z}\right)^{\frac{\alpha}{\alpha-1}} \\ &\ge \beta\Big(1-\theta(\delta^*_{1,RV}, 1)\Big)\pi + \Big[\frac{N_1\cdot 1}{\alpha} + \beta\Big(1+\frac{1}{\alpha} - \theta(\delta^*_{1,RV}, 1)\Big)\big(N_1 - 1 + \theta(\delta^*_{1,RV}, 1)\big)\Big] Z(1-\alpha)\alpha^{\frac{1+\alpha}{1-\alpha}}\left(\frac{\pi}{z}\right)^{\frac{\alpha}{\alpha-1}} \\ &= T^C_{MN} \end{aligned}$$

where the inequality follows by the fact that $\ell^*_{1,RV}$ is the civilian's best response given $\delta^*_{1,RV}$. Being trapped in conflict, however, the elite is worse off,

$$\begin{aligned} T^E_{RV} &= (1-\delta^*_{1,RV} + \beta\theta(\delta^*_{1,RV}, \ell^*_{1,RV}))\pi + \Big[(1-\delta^*_{1,RV})N_1\ell^*_{1,RV} + \beta\theta(\delta^*_{1,RV}, \ell^*_{1,RV})N_2\Big] Z\frac{1-\alpha}{\alpha}\alpha^{\frac{2}{1-\alpha}}\left(\frac{\pi}{z}\right)^{\frac{\alpha}{\alpha-1}} \\ &\le T^E_{MN} \end{aligned}$$

All the inequalities from (S-20) through (S-22) become strict when conflict prevails, i.e., $\ell^*_{t,RV} < 1$ and thus $\delta^*_{t,RV} > 0$ by Proposition S-1.

*The IM model*: Recall that in this model, there is no social fractionalization and all resources are imported. There is no doubt that labor is fully supplied by the civilian, i.e.,

$$\ell^*_{t,IM} = 1 \tag{S-23}$$

By maximization, the domestic consumption of resource goods satisfies

$$\sigma^*_{t,IM} = \left(\frac{\pi}{\alpha Z}\right)^{\frac{1}{\alpha-1}} N_1 \tag{S-24}$$

which implies the equilibrium domestic production

$$F_{t,IM} = Z\left(\frac{\pi}{\alpha Z}\right)^{\frac{\alpha}{\alpha-1}} N_1 \tag{S-25}$$

and the civilian's total payoff

$$T^C_{IM} = (1+\beta)Z(1-\alpha)\alpha^{\frac{\alpha}{1-\alpha}}\left(\frac{\pi}{z}\right)^{\frac{\alpha}{\alpha-1}} N_1 \tag{S-26}$$

We claim that there are cases in which the civilian of a resource-abundant economy is poorer than that of a resource-deficient economy due to market monopoly and civil conflict:

**Lemma S-13.** *For any world resource price $\pi$, let $T^C_{RV}(\pi), T^C_{IM}(\pi)$ denote the civilian's total expected payoffs at equilibria of the two models $RV$ and $IM$. Let the conflict technology, i.e, $\mu, \gamma$, be constant. If either $\alpha$ or $\beta$ is sufficiently low, then there exists a $\underline{\pi} \in (P^*, \infty)$ such that*

$$T^C_{RV}(\pi) < T^C_{IM}(\pi), \quad \forall \pi \in (\,P^*, \underline{\pi}\,]$$

*Proof.* Note that we have

$$
\begin{aligned}
&T^C_{RV}(\pi) - T^C_{IM}(\pi)\\
&\leq (1+\beta)Z(1-\alpha)\alpha^{\frac{2\alpha}{1-\alpha}}\left(\frac{\pi}{z}\right)^{\frac{\alpha}{\alpha-1}} N_1 + \beta\left(\pi + Z(1-\alpha)\alpha^{\frac{1+\alpha}{1-\alpha}}\left(\frac{\pi}{z}\right)^{\frac{\alpha}{\alpha-1}} N_1\right) - (1+\beta)Z(1-\alpha)\alpha^{\frac{\alpha}{1-\alpha}}\left(\frac{\pi}{z}\right)^{\frac{\alpha}{\alpha-1}} N_1
\end{aligned}
$$

Denote $\Gamma(\pi) = T^C_{RV}(\pi) - T^C_{IM}(\pi)$. We claim that $\Gamma$ is increasing on $[P^*, \infty)$. To see this, note that for all $\pi \in (P^*, \infty)$,

$$
\begin{aligned}
\Gamma'(\pi) &= -(1+\beta)Z\alpha^{\frac{1+\alpha}{1-\alpha}} Z^{\frac{\alpha}{1-\alpha}} \pi^{\frac{1}{\alpha-1}} N_1 + \beta - \beta Z\alpha^{\frac{2}{1-\alpha}} Z^{\frac{\alpha}{1-\alpha}} \pi^{\frac{1}{\alpha-1}} N_1 + (1+\beta)Z\alpha^{\frac{1}{1-\alpha}} Z^{\frac{\alpha}{1-\alpha}} \pi^{\frac{1}{\alpha-1}} N_1\\
&> \beta - \beta Z\alpha^{\frac{2}{1-\alpha}} Z^{\frac{\alpha}{1-\alpha}} \pi^{\frac{1}{\alpha-1}} N_1\\
&> \beta - \beta Z\alpha^{\frac{2}{1-\alpha}} Z^{\frac{\alpha}{1-\alpha}} (P^*)^{\frac{1}{\alpha-1}} N_1\\
&> 0
\end{aligned}
$$

Hence, our claim follows by Lemma S-1.

Additionally, we have

$$\lim_{\pi\to\infty} \Gamma(\pi) = \infty$$

and

$$\Gamma(P^*) = \left((1+\beta)(1-\alpha)\alpha^{\frac{\alpha}{1-\alpha}} + \alpha\beta + \alpha\beta(1-\alpha)\alpha^{\frac{\alpha}{1-\alpha}} - (1+\beta)(1-\alpha)\right) Z N_1^{1-\alpha}$$

If either $\alpha$ or $\beta$ is sufficiently small, it is not difficult to see that $\Gamma(P^*) < 0$. Consequently, the lemma follows by the continuity and monotonicity of $\Gamma$. □

Altogether, we have proved Theorem 2.

# References


1. Bartle R, Sherbert D (1999) Introduction to Real Analysis. New Jersey: Wiley. 169, 188 p.

2. Fudenberg D, Tirole J (1991) Game Theory. Cambridge: Massachusetts Institute of Technology Press. 72-74 p.

3. Osborne M (2004) An Introduction to Game Theory. New York: Oxford University Press. 172 p.
